# Patient advocate involvement in the design and conduct of breast cancer clinical trials requiring the collection of multiple biopsies

**DOI:** 10.1186/s40900-018-0108-0

**Published:** 2018-07-16

**Authors:** Leona M. Batten, Indrani Subarna Bhattacharya, Laura Moretti, Joanne S. Haviland, Marie A. Emson, Sarah E. Miller, Monica Jefford, Mairead MacKenzie, Maggie Wilcox, Marie Hyslop, Rachel Todd, Claire F. Snowdon, Judith M. Bliss

**Affiliations:** 1 Division of Clinical Studies, The Institute of Cancer Research Clinical Trials and Statistics Unit, Sir Richard Doll Building, 15 Cotswold Road, Sutton, London, SM2 5NG UK; 2 National Cancer Research Institute, Angel Building, 407, St John Street, Clerkenwell, London, EC1V 4AD UK; 3 Independent Cancer Patients Voice, 17 Woodbridge St, Clerkenwell, London, EC1R 0LL UK

**Keywords:** Biopsy, Multiple biopsies, Patient advocate, Clinical trial, Breast cancer, Neoadjuvant, Aromatase inhibitor

## Abstract

**Plain English summary:**

Breast cancer is a diverse and varied disease. Recent research has shown that the collection of multiple biopsies before surgery can help researchers determine how the cancer is responding to treatment and can predict for long-term outcomes. However biopsies can be uncomfortable, and sometimes clinicians and research teams in hospitals may be reluctant to offer clinical trials requiring several biopsies to patients who have been recently diagnosed with breast cancer. The Institute of Cancer Research Clinical Trials and Statistics Unit (ICR-CTSU) oversees a large number of breast cancer clinical trials where multiple biopsies are required. ICR-CTSU recognises that patient advocates (patients who have previously had, or cared for someone with, cancer) are key members of the trial design group and should be involved in the clinical trial throughout its lifespan. Patient advocates can provide reassurance regarding the acceptability of trial designs involving multiple biopsies from a patient perspective. This paper summarises patient advocate involvement in ICR-CTSU breast cancer trials activity and how this has benefited our research.

**Abstract:**

The importance of collecting tissue samples in breast cancer has become increasingly recognised, as the diversity of the disease has become better known. It has been documented in recent research that tumours may change in response to treatment prior to surgery (the neoadjuvant treatment setting). The collection of sequential biopsies over time can identify changes within tumours and potentially predict how the tumour may respond to certain treatments. However, the acceptability of multiple biopsies amongst patients, clinicians and other research staff in hospitals is variable and recruitment into clinical trials requiring multiple biopsies may be challenging.

The Institute of Cancer Research Clinical Trials and Statistics Unit (ICR-CTSU) is responsible for a portfolio of breast cancer trials where multiple biopsies are key to the trial design. Patient advocate involvement has been essential in helping us to design and deliver complex and innovative cancer trials which require multiple invasive tissue biopsies, often without any direct benefit to the trial participants. The views expressed by patient advocates involved in ICR-CTSU trials supports the published evidence that patients are willing to donate additional tissue for research and that clinicians’ concerns about approaching patients for trials involving multiple biopsies are often unfounded.

Patient advocate involvement in ICR-CTSU trials activity takes various forms, from membership on protocol development groups and trial management groups, attendance at focus groups and forums, and presentations at trial development and launch meetings. This involvement has provided reassurance to research teams within the NHS and research ethics committees of the importance and acceptability of our trials from a patient perspective. Patient advocate involvement throughout the lifetime of our trials ensures that the patient remains central to our research considerations.

## Background

In recent years, breast cancer has been recognised as a diverse and varied disease consisting of many different subtypes [1–3]. Collecting a tissue sample (or biopsy) from a patient is not only essential for confirmation of the diagnosis of breast cancer but also has the potential to define the breast cancer subtype in order to inform treatment choices. This is achieved by the identification of mutations (changes to normal genetic information) that are present in the tumour tissue sample. An example of this is the established use of aromatase inhibitor therapy in the treatment of postmenopausal women with oestrogen receptor positive breast cancer. Aromatase inhibitors work by blocking the enzyme aromatase, which turns the hormone androgen into small amounts of oestrogen in the body. This means that less oestrogen is available to stimulate the growth of hormone receptor positive breast cancer cells.

The pre-surgical (neoadjuvant) treatment setting is increasingly being used to provide early evidence of the clinical activity of new treatments. In particular the assessment of tumour tissue obtained during neoadjuvant treatment from biopsies taken prior to surgery (i.e. while the tumour remains ‘in situ’) can aid the identification of biological markers, which provide a measurable indicator of response and resistance to treatment. The biological analysis of sequential biopsies obtained before, during and after treatment can reveal how the cancer changes over time and responds to the treatment. Therefore, obtaining additional breast cancer tissue during treatment has become an integral component in breast cancer research [4]. Knowing which patient population may be responsive to treatment and which biological markers may indicate resistance has the potential to reduce the risk of patient exposure to treatment that is unlikely to be of benefit, and increase the likelihood of patient exposure to active treatment.

Undergoing a biopsy is an invasive procedure which can result in additional discomfort and inconvenience for the patient and can be technically challenging due to the site of the disease. One of the main challenges faced in the collection of multiple tissue biopsies is the perception of clinicians that patients may not wish to be approached for the additional biopsies, or they may not want to subject patients to additional procedures that would not be routinely performed as part of standard of care. The reasons cited for not wanting to approach patients for additional biopsies include the risk associated with the biopsy procedure, the pain or discomfort of a biopsy and the inconvenience to patients [4]. Whilst studies have shown that patients are willing to provide research biopsies even when there is no direct benefit to them [5–9], treating clinicians and other research staff may remain reluctant due to perceived ideas of what is acceptable.

Ethical concerns have also been raised about the collection of research biopsies that will not directly benefit the patient [9–11]. The risks associated with taking research biopsies have been shown to be more acceptable to patients than research ethics committees (RECs) who may consider that the benefits of acquiring additional tissue from trial participants purely for research may not outweigh the risks [8, 9]. The importance of well written patient information sheets that clearly describe the risks and lack of direct benefit associated with research biopsies is well documented [12, 13].

Involving patient advocates in the concept development, design and conduct of a clinical trial is important [14–16], particularly in trials perceived as challenging for the patient such as those requiring multiple biopsies. The importance of patient advocate involvement throughout the whole clinical trials process has long been recognised by ICR-CTSU, a clinical trials unit based in London that coordinates national and international phase II and III clinical trials with a particular focus on breast cancer. ICR-CTSU has had close links to several patient advocate groups for several years. Their input has been crucial in developing participant information materials that effectively describe the research and the associated risks and benefits in an accessible language that addresses the recognised concerns of patients with a cancer diagnosis being required to provide multiple biopsies. They have also provided reassurance to clinicians, research nurses and RECs on the acceptability of the collection of multiple biopsies from a patients’ perspective.

Patient advocates review and advise on all ICR-CTSU trials, and there is routinely at least one patient advocate member on the trial management group (a multidisciplinary group responsible for day to day oversight of the trial) for each trial. This ensures that patient advocates have ongoing oversight of the clinical trials and are able to give recommendations and advice throughout the lifespan of the trial.

In this article we discuss the processes undertaken by ICR-CTSU to optimise patient advocate engagement across a number of our trials requiring the collection of multiple biopsies and how this has ensured successful implementation and delivery of our research.

## Main text

Change in the proliferation marker Ki67 (a marker of a rapid increase in cell numbers as measured on tumour tissue) has been shown to be predictive of response to treatment with aromatase inhibitors and can be used as an indicator of risk of breast cancer occurring again (recurrence). Patients in the Immediate Preoperative Anastrozole, Tamoxifen, or Combined with Tamoxifen (IMPACT) trial [17, 18], conducted by colleagues at the Royal Marsden Hospital, had oestrogen receptor positive early breast cancer and were randomised to receive aromatase inhibitor therapies (either anastrozole alone, tamoxifen alone or anastrozole and tamoxifen in combination). Patients had biopsies taken at baseline, at 2 weeks after starting treatment and at surgery (approximately 12 weeks after starting treatment). The trial recruited 330 patients from 1997 to 2002, and patients were followed up for several years. Data from the sequential biopsies showed that short-term changes in Ki67 may be able to predict disease outcomes.

On the basis of results from the IMPACT trial Ki67 has since been used as the primary efficacy end point providing the potential to use short-term molecular markers to predict long-term outcome in clinical trials to allow effective treatments to reach patients more rapidly than waiting for long-term measures [19].

### The POETIC trial

Following on from the IMPACT trial, the ICR-CTSU in collaboration with the Royal Marsden Hospital, designed the POETIC (Perioperative Endocrine Therapy - Individualising Care, ISRCTN: 63882543) trial [20], a randomised controlled trial investigating aromatase inhibitor therapy in oestrogen positive early breast cancer prior to and after surgery (the perioperative treatment setting). The trial design required a research biopsy taken at diagnosis with a subsequent biopsy after 14 days treatment with an aromatase inhibitor prior to definitive surgery for assessment of Ki67 expression. Concerns were raised about the concept of taking invasive research biopsies in this relatively low risk patient population and how this would be viewed by patients, their treating clinicians and breast care nurses so involvement of patient advocates was essential.

Patient advocates were involved in the trial design from the outset with an extensive consultation process being undertaken across a number of patient groups. They were consulted on the fact that research tissue collected at diagnosis may be provided by patients who subsequently turned out to be ineligible for trial entry, and that collecting research tissue at that time point would allow little time for patients to consider whether they wanted to donate tissue for research purposes. The patient advocates provided reassurance that requesting additional samples from the patient was acceptable providing the collection process was led by a skilled team, the rationale for collecting the additional tissue was clearly explained to the patient and donated samples were utilised appropriately. They recommended that extra tissue for research at diagnosis was collected at the same time as the routine core biopsy, and not after diagnosis was confirmed. They also preferred any tissue taken to be used for research wherever possible. This provided valuable insight to assist communications with the REC about the proposed research [16].

Patient advocates advised on the content of the patient information sheets for the trial to ensure that the research rationale, procedures and risks were fully explained and in an accessible language. A patient advocate was a member of the protocol development group and, once the trial was launched, a member of the trial management group, providing advice throughout the lifetime of the trial. Once launched, the trial met with some reluctance, mainly from breast cancer nurses, about approaching patients for participation in the trial. Intervention from patient advocates via teleconferences and newsletters conveyed the message that professionals should not avoid discussing participation with potential patients at the time of diagnosis due to concern about adding stress, as this denies patient choice.

Despite the fact that patients had only recently received their diagnosis, willingness to undergo additional procedures and consider clinical trial participation was high in POETIC and recruitment into the trial remained on target. POETIC recruited 4486 patients between 2008 and 2014 and provides the largest multi-centre series of women in whom centrally assessed Ki67 has been correlated with classic clinical and pathological measures of response. When the paired research biopsies from each patient were compared, 29% of patients were found to have mutations present in only one of their two biopsies [21]. POETIC showed that for a substantial group of patients, a single biopsy would have missed potentially important mutations that may have influenced the treatment options for the patient. The variation in the mutations identified between the paired biopsies may be due to the genetic differences between the different areas of the tumour that were sampled but also may be due to changes to the tumour cells in response to treatment given. Irrespective of the reason, this finding led the POETIC investigators to conclude that multiple biopsies are essential for confident mutational profiling of oestrogen receptor positive breast cancer. POETIC demonstrated that patients are willing to discuss and consider clinical trials which request additional biopsies whilst they are coming to terms with their diagnosis. It also shows that education regarding the value and purpose of the collection of multiple biopsies is essential for both clinicians and patients.

### The PALLET trial

Subsequent to POETIC, ICR-CTSU in collaboration with colleagues at the Royal Marsden Hospital, designed the PALLET trial (An assessment of the biological and clinical effects of palbociclib with letrozole in the neoadjuvant treatment of ER+ primary breast cancer, ISRCTN: 31243262). PALLET is a randomised phase II trial in early breast cancer aiming to investigate whether the addition of palbociclib (a targeted drug that blocks CDK4/6 proteins involved in cell division) to letrozole (an aromatase inhibitor) in the neoadjuvant setting (prior to surgery) can reduce tumour size and assess the effect of treatment on the reduction of tumour cell proliferation by measuring Ki67 at multiple time points. The assessment of treatment effect required participants consent to provide a biopsy at the time of trial entry, and then at 2 weeks and 14 weeks after trial entry. Up to 4 samples were taken using a core needle biopsy at each of the 3 time points. Based on our experiences with POETIC it was recognised that clinicians and breast nurses may consider the additional required biopsy procedures too onerous for patients and may not approach patients for the trial, so involvement of the patient advocates from the outset was crucial.

Patient advocates from Independent Cancer Patients Voice (ICPV), who had contributed so positively to POETIC, and from the Royal Marsden Patient and Carers Research Review Panel were approached for collaboration and involvement in the PALLET trial. The patient advocates were involved during all stages of the trial including conception and trial launch and have an ongoing role as members of the trial management group. During the development and set-up of PALLET, the patient advocates advised that patients would consider entering the trial provided the rationale for the multiple biopsies was clear in the patient information documentation and there was detailed information regarding the biopsy process, including potential risks and post-biopsy care. The patient advocates advised that patients often felt reassured by having additional investigations for research as part of their participation in a clinical trial, provided these were clearly explained upfront and that patients commonly felt better informed of their breast cancer disease as a result of involvement in clinical trials. The PALLET patient information sheet was reviewed by the patient advocates prior to submission to the REC to ensure all relevant and useful information was included for the committee to make an informed assessment of the benefits and risks associated with the trial. One concern raised by the REC which had previously been raised by the patient advocates was around the potential risk of seeding (spread of tumour cells into the blood or other tissue) as a result of having a biopsy. The lead investigator for the trial was able to provide reassurance to the patient advocates about the minimal risks of seeding which the patient advocates found acceptable. This in turn provided reassurance to the REC.

In order to minimise the potential impact that any pre-conceptions around patient acceptance may have had on the rate of recruitment into the trial, patient advocates from ICPV gave a presentation at the PALLET trial launch meeting. They were able to draw on their experiences from the POETIC trial, particularly the consultation process that had taken place across a number of patient advocate groups around the acceptability of research biopsies from a patient perspective, and were able to provide reassurance the national investigators and breast cancer research nurses in attendance. An outline of the points raised by the patient advocates at the trial launch meeting is summarised in Table 1.

**Table 1:** Patient concerns regarding trials involving multiple biopsies

Patient concern	More likely to accept	Less likely to accept
Comfort and safety	• Accessible biopsy site • Good post-biopsy care offered • Collection of research tissue during standard procedures	• Challenging biopsy site • Post-biopsy care not explained • Additional visits or procedures for tissue collection
Risks and benefits	• Given sufficient information about purpose of the research and need for biopsies • Risks of tumour seeding explained • Other risks, including discomfort at biopsy site, fully explained • Lack of direct benefit fully explained • Research is considered ‘exciting’ – involving and educating patients • Research will improve outcomes in future patients and exclude unnecessary treatment in future patients	• Not given sufficient information about purpose, risks and benefits
Timing	• Given adequate time to decide	• Badly timed or insensitive introduction to trial
Quality control	• Clear explanation of what happens to the samples	• Use of samples not adequately explained
History	• Patients with metastatic disease • Family history	• Previous poor experiences of research

Twelve months after opening the first site to the PALLET trial, the recruitment rate was lower than expected for a variety of reasons ranging from logistical issues at sites to insufficient numbers of patients being approached for the trial. It became apparent that a major recruitment barrier was that clinicians were either not approaching suitable patients, or not discussing the trial adequately or at an appropriate time. For example, if clinicians offered surgery dates prior to offering a clinical trial of neoadjuvant treatment, patients often declined to take part as they didn’t want to ‘delay’ their surgery by having the neoadjuvant treatment. It was observed that not approaching patients for the trial had a greater impact on recruitment than patients declining due to the requirement for multiple research biopsies. In response to this, a teleconference was arranged for the clinicians and research nurses at recruiting centres, and the PALLET trial management group patient advocates led a discussion on the reasons clinicians may be reluctant to approach patients for the trial. The patient advocates re-iterated that it’s ok to ask patients about entering clinical trials including those involving multiple biopsies. They highlighted that the requirement for additional biopsies tended not to discourage patients from participating in a clinical trial, provided the reason was clearly explained and the biopsies were performed in the appropriate clinical setting with good post biopsy care provided. The patient advocates referred to the fact that clinicians often believed that patients would be deterred from entering a trial requiring multiple research biopsies to a much greater extent than they actually were. The importance of offering all potential treatment options, including clinical trials, to patients was highlighted in order that patients can make their own informed decision about their preferred treatment choice, rather than being limited to what their clinician thought was best for them. Following the teleconference, the rate of recruitment into PALLET improved and the trial reached its target recruitment size within 3 years of the first site opening.

### Multiple biopsies patient advocate forum

Following on from the issues identified in POETIC and PALLET, and to inform a number of new breast cancer trials in development, ICR-CTSU arranged a Patient Advocate Forum to facilitate discussion between a wide audience of patient advocates and clinical investigators regarding the development of clinical trials requiring the collection of multiple biopsies. Clinicians and research nurses working in breast cancer research were invited to attend, in addition to members from national patient and public involvement groups including those who had been involved in the design and conduct of POETIC and PALLET. A number of new trial concepts requiring multiple biopsies were presented and discussed.

The patient advocates emphasised that it is important that patients are provided with all the different treatment options available to them, including clinical trials where multiple biopsies are required. They reiterated that patients should be fully informed about the trial design, the potential risks and benefits of participating in the trial and it should be up to the patient to decide if it is a trial they would like to participate in based on the information provided. Whilst the patient advocates did not think the requirement for multiple biopsies within a trial would be a barrier to trial entry from a patient perspective, they advised that, where possible, researchers should ensure that any additional biopsies are performed on the same day as other trial assessments to minimise the need for patients to arrange, and pay for, additional travel. It was also noted that there should be an adequate gap between the sequential biopsies for patient recovery. Attendees reiterated that it should be up to the patient to make a final decision on their treatment pathway once all information has been provided to them, re-emphasising the need for better clinician training in this area.

The forum included a brief presentation on the potential future use of circulating tumour DNA (ctDNA) measured in the blood. As ctDNA may contain mutations present in the tumour that aren’t present in the patient’s normal genetic information, ctDNA testing could be used as an alternative ‘liquid biopsy’ in order to identify the mutations present in the cancer and direct treatment whilst reducing the need for an invasive tissue biopsy. The importance of identifying a less invasive alternative to tissue biopsies is well recognised [9, 22]. Liquid biopsies may be particularly beneficial to reassess the mutations present when the cancer spreads to another part of the body (relapse, or metastases) and is not easily accessible for biopsy. This is important as clinicians are more reluctant to approach patients for biopsies of metastatic disease sites [4, 6, 22]. However, in order to evaluate the use of ctDNA as a screening tool there is a need to compare liquid biopsy results with the results obtained from a tissue sample biopsy to ensure the ctDNA screening is reliable and accurate. Clinical trials are currently being conducted to address this before the use of ctDNA testing could become routine practice in the future.

Feedback from the meeting was used in initial applications to, and during discussions with, regulators and RECs regarding the proposed trial designs that utilise multiple tissue or liquid biopsies. Patient advocate support for these trials undoubtedly provided reassurance to regulators and RECs regarding the importance and acceptability of these novel trial designs to patients.

## Conclusions

As breast cancer treatments become more specific, or ‘personalised’, the inclusion of multiple biopsies in the design of clinical trials is becoming more common [4, 8, 9]. The collection of multiple biopsies allows the identification of changes in the molecular profile of the disease over time and in response to treatment received. Patients are now frequently required to consent upfront to the collection of additional research biopsies during trial participation. Despite patient willingness to enter trials that require multiple biopsies [6–8, 23] concerns exist amongst clinicians and research nurses about approaching patients due to misconceptions around patient acceptance of potentially uncomfortable and inconvenient research procedures [4, 6, 8, 9]. Ethical concerns around the risks of the biopsy procedure and lack of direct benefit to the patient exist [8–12, 22]. Patient advocate involvement can help allay these concerns and provide reassurance about the acceptability of multiple biopsies from a patient perspective.

Patient advocates have emphasised that when approaching patients about trials requiring multiple biopsies, it is of paramount importance to describe the rationale behind the collection of multiple biopsies and the associated risks. Well written patient information sheets enable patients to make an informed assessment of the risks and benefits; patient advocate involvement in the development of these materials is essential to ensure that the information is conveyed in an accessible and understandable format. The use of teleconferences with participating hospitals which include involvement of the patient advocates and patient advocate representation at trial launch meetings can help to provide hospital staff at recruiting sites with a clearer idea as to the patient perspective on trials requiring multiple biopsies.

Further education and communication in the area of multiple biopsies is required amongst clinicians and care teams, to ensure patients are introduced to the trials appropriately, sensitively, and to minimise clinicians’ discomfort with the idea of obtaining sequential biopsies from patients throughout the lifespan of a clinical trial. The use of novel less-invasive techniques, such as liquid biopsies, may provide a more acceptable alternative to clinicians and patients alike in the future. Forums organised to facilitate discussion between clinicians and patient advocates can help address concerns about new trial designs, including those involving multiple biopsies, and new techniques, and provide an excellent learning opportunity for all involved.

ICR-CTSU has a long history of working with patient advocates [16] and has a strong belief that researchers should employ a more consistent, focused approach when involving patient advocates in the development of clinical trials. This is now a requirement of many funders and RECs. Well-organised patient advocate involvement can help to increase the success of a clinical trial providing RECs, regulators, clinicians and research nurses with reassurance about the acceptability of trial procedures, ultimately ensuring successful recruitment into the trial. We recommend that patient advocates are involved from the trial design stage and throughout the lifetime of the trial through membership on protocol development and trial management groups. Feedback from researchers to patient advocate groups is also important. Our experience is that patient advocates appreciate understanding how their input has benefitted the trial and are more likely to engage in the development of future trials as a result.

Patient advocate groups are a key link between researchers and the general public whose consent to participation in clinical trials is crucial to their success. The need for patients to be informed of all options and the reasons behind trial requirements, such as multiple biopsies, is essential and should not be overlooked. The involvement of patient advocates throughout clinical trials from design to publication is essential and should be taken into consideration from the point of trial conception.

## Abbreviations

ctDNA: Circulating tumour DNA
ICPV: Independent Cancer Patients’ Voice
ICR-CTSU: Institute of Cancer Research Clinical Trials and Statistics Unit
IMPACT: Immediate Preoperative Anastrozole, Tamoxifen, or Combined with Tamoxifen trial
NIHR: National Institute for Health Research
PALLET: An assessment of the biological and clinical effects of palbociclib with letrozole in the neoadjuvant treatment of ER+ primary breast cancer
POETIC: Perioperative Endocrine Therapy - Individualising Care trial
REC: Research Ethics Committee
